# Changes in Urinary Microalbumin Levels after Correction of Hyperuricemia in Patients with Gout: An Observational Cohort Study

**DOI:** 10.1155/2020/8310685

**Published:** 2020-04-01

**Authors:** Binit Vaidya, Kalpana Pudasaini, Shweta Nakarmi

**Affiliations:** National Center for Rheumatic Diseases (NCRD), Kathmandu, Nepal

## Abstract

**Background:**

Gout is commonly associated with metabolic syndrome. Strong association between the serum uric acid level and microalbuminuria has also been observed in various studies.

**Aim:**

To observe the change in urinary microalbumin after urate-lowering treatment in patients with gout and microalbuminuria. *Methodology*. A prospective, observational study was conducted at a tertiary-level rheumatic center (NCRD) in Kathmandu, Nepal. Adults diagnosed with gout using the 2015 ACR/EULAR criteria and microalbuminuria were enrolled in the study after obtaining informed consent. Sociodemographic profile and clinical history were recorded at baseline. Serum uric acid levels, spot urinary microalbumin (MAU) excretion, blood sugar, lipid profile, and blood pressure were measured at baseline, 3-month follow-up, and 6-month follow-up. A paired *t*-test was used to compare the change in mean MAU after treatment.

**Results:**

A total of 778 patients diagnosed with gout were screened for microalbuminuria. Among them, 114 (14.6%) had urinary microalbumin levels of >30.0 mg/L during presentation. Mean MAU level among those with microalbuminuria was 132.4 ± 124.6 mg/L. Thirty-five patients had concomitant HTN and were put on ARBs (20 mg of telmisartan). All received 40 mg of febuxostat. In patients with ARBs, MAU reduced significantly after 3 months of treatment with ARBs. Reduction in MAU in those without ARBs was seen after the 6-month follow-up, and the change was statistically significant.

**Conclusions:**

There is significant reduction in MAU after the use of urate-lowering drugs in patients with gout.

## 1. Introduction

Gout is increasingly recognized as one of the major health problems in recent decades [1]. It is usually associated with an elevated serum uric acid (SUA) level. Higher SUA levels above the saturation threshold tend to form and deposit monosodium urate (MSU) crystals in and around joints [2]. Uric acid is a purine metabolic product excreted mainly by kidneys (2/3^rd^ amount) [3]. Apart from hyperuricemia, several risk factors for development of gout have been postulated in different studies including genetic factors, dietary factors, alcohol consumption, diuretic use, and renal disease [4]. There are studies showing the close association of hyperuricemia or gout with metabolic syndrome [5–7]. Gout can be considered as a risk factor for hypertension (HTN), cardiovascular disease, chronic kidney disease (CKD), and diabetes [8]. Few studies suggest that uric acid contributes to increased inflammation leading to CKD progression, thus demonstrating the relationship between serum urate levels and kidney function. While hyperuricemia can cause the progression of renal disease, kidney dysfunction itself can increase serum urate levels due to glomerular damage leading to reduced excretion of serum uric acid [6, 7].

Microalbuminuria (MAU) is defined as the persistent elevation of albumin in urine ranging from 30 to 300 mg/day in a spot urine sample [9]. MAU is an established marker of risk calculation for the presence of cardiovascular disease and also predicts the progression of nephropathy when its level peaks above 300 mg/day, reflecting generalized endothelial damage [10, 11]. Allopurinol and febuxostat are the frequently used urate-lowering agents (ULT) which cause competitive inhibition of xanthine oxidase [12]. Although some of the studies have suggested association between these ULTs (febuxostat and allopurinol) and kidney function, only few studies have reported the effect of urate-lowering therapy in preventing deterioration of kidney function or reduction in microalbuminuria [13, 14]. Change in the serum uric acid level and the urinary microalbumin level with treatment of gout is yet to be assessed clearly. This study was aimed at studying the prevalence of microalbuminuria in gout patients and the effect of urate-lowering therapy on urinary microalbumin levels.

## 2. Materials and Methods

### 2.1. Ethics Statement

This was a prospective, observational, cohort study conducted at the National Center for Rheumatic Diseases (NCRD), a tertiary-level rheumatology center in Nepal. Written informed consent from each patient was obtained, and ethical approval was taken from the institutional review board.

### 2.2. Study Population

Consecutive patients diagnosed with gout based on the 2015 ACR/EULAR criteria [15] attending the rheumatology clinic at the National Center for Rheumatic Diseases, Kathmandu, Nepal, from June 2016 to May 2019 were screened for microalbuminuria. Patients with severe cardiac, renal, neurological, and other inflammatory diseases and those who are receiving or have received urate-lowering therapy, angiotensin-converting enzyme inhibitor (ACE-I), or angiotensin receptor blocker (ARB) in the last 3 months were excluded. Pregnant and lactating mothers were also excluded from the study.

### 2.3. Treatment Groups

#### 2.3.1. ARB Group

Patients with microalbuminuria and concomitant HTN were assigned to ARB (20 mg of telmisartan, once daily) and febuxostat.

#### 2.3.2. Non-ARB Group

Those with microalbuminuria but no concomitant HTN were given febuxostat only. The dose of urate-lowering therapy (febuxostat) was initiated at 40 mg once a day and was titrated to lower serum urate levels to 4 to 6 mg/dL.

### 2.4. Collection of Data

Sociodemographic profile and clinical data were recorded in a predesigned Excel sheet at baseline. Serum uric acid level, spot urinary microalbumin level, blood sugar level, lipid profile, serum creatinine, and blood pressure were measured at baseline, 3-month follow-up, and 6-month follow-up. HTN was defined as systolic BP ≥ 140 mmHg and/or diastolic BP ≥ 90 mmHg and/or current use of antihypertensive medication [16]. Likewise, microalbuminuria was defined as a spot urinary albumin level between 30 and 300 mg/L [9].

### 2.5. Laboratory Methods

SUA was measured by the enzymatic uricase method using an automated analyzer (Erba XL 200; Erba Diagnostics, Mannheim, Germany). Urine microalbumin was measured by antigen antibody reaction, using an automated analyzer (Erba XL 200; Erba Diagnostics, Mannheim, Germany). Blood pressure was measured using a sphygmomanometer.

### 2.6. Statistical Analysis

Statistical analysis was carried out with the help of SPSS software (version 16.0 system for Windows (SPSS, Chicago, IL)). A paired *t*-test was performed to compare the difference between pre- and post treatment mean serum uric acid levels and urinary microalbumin levels within the group. An independent *t*-test was done to compare mean serum uric acid levels and urinary microalbumin levels in between the two groups. All *p* values presented were two-tailed, and a value of 0.05 or less was considered as statistically significant.

## 3. Results

Out of 778 patients with gout screened, 14.6% patients showed microalbuminuria during presentation. The mean age of 114 patients with microalbuminuria was 51.1 ± 12.6 years with mean BMI of 27.0 ± 5.3 kg/m^2^. The mean MAU level among those with microalbuminuria was 132.4 ± 124.6 mg/L. Among the 114 patients with high MAU, 35 patients were found to have concomitant HTN and were kept on telmisartan. Other clinic-demographic profiles of the two study groups (ARB and non-ARB) are shown in Table 1.

Serum UA levels decreased to the target level of 4-6 mg/dL in both groups post treatment. There was no significant difference in UA levels at the 3- and 6-month follow-ups in between the 2 treatment groups as shown by the independent *t*-test (*p* values 0.576 and 0.219 for the 3- and 6-month follow-ups, respectively). In patients on ARB, the mean MAU levels at baseline, 3-month follow-up, and 6-month follow-up were 127.9 ± 111.6, 72.0 ± 109.3, and 44.9 ± 61.6 mg/L, respectively. The reduction in MAU in this group was statistically significantly after 3 months of treatment with ARBs (Table 2). In the patients without ARB, the mean MAU levels at baseline, 3-month follow-up, and 6-month follow-up were 69.6 ± 14.6 mg/L, 42.2 ± 54.4 mg/L, and 15.7 ± 4.9 mg/L, respectively. Significant reduction in MAU in those without ARB was seen after the 6-month follow-up (Table 3).

## 4. Discussion

Gout is a common rheumatic disorder with a prevalence of 0.1-10% of the general population with higher prevalence in the male gender before the menopausal age of females [17]. It is characterized by hyperuricemia and deposition of urate crystals in the tissues, of which the most common is the synovium [2, 18, 19]. However, only 5% of patients with hyperuricemia develop gout [2]. It has been seen that the disorder, either gout or hyperuricemia, has been associated with derangement of various metabolic parameters like microalbuminuria, dyslipidemia, nonalcoholic steatohepatitis, chronic kidney disease, and cardiovascular diseases [20–24].

The relationship between uric acid and the urinary albumin excretion rate is a well-known fact. Increased prevalence of microalbuminuria with or without CKD in patients with gout or hyperuricemia has been seen [25]. In a study done in rats by Mazzali et al., it was shown that even mild hyperuricemia can cause renal injury and albuminuria [26]. Also, many authors have focused on the relationship between albuminuria and SUA documenting positive association between them in the general population and in patients with diabetes mellitus and hypertension [27–29]. It seems that as increased serum uric acid predisposes to nephropathy and loss of kidney function, lowering its levels can be helpful in preventing it. In a murine study, induced hyperuricemia resulted in HTN, renal arteriolopathy along with glomerular HTN with tubulointerstitial inflammation, and fibrosis. When hyperuricemia was corrected to normal levels with urate-lowering agents, the above-mentioned manifestations were all corrected [26, 30–32].

Urate-lowering therapy leads to suppression of the renin-angiotensin-aldosterone axis, improves nitric oxide bioavailability as well as endothelial function, and reduces the oxidative stress and urinary inflammatory biomarkers [30, 33–35]. It has been reported that kidney function improved by treatment with allopurinol or febuxostat for gout [13, 36]. Our observation suggests that treatment of gout with ULT can result in improvement of associated microalbuminuria. Similar observations were made in other studies. In a study by Ueno, kidney function was ameliorated and albuminuria was decreased in patients with gout on ULT suggesting that ULT could prevent kidney function if started as early as possible [37]. Pai et al. found an interesting observation that allopurinol was effective in reducing uric acid and preventing renal dysfunction, hypertension, proteinuria, renal hypertrophy, vascular disease, and renal scarring [38]. Another drug, benziodarone, a uricosuric agent, however, did not account for any of the above-mentioned benefits, and lowering of uric acid was less effective compared to allopurinol and febuxostat in a previous study strengthening the hypothesis [39].

In our study, we could observe that the serum uric acid level was not dramatically reduced in either group with the mean level remaining more than 5 mg/dL, though the change reached the level of statistical significance compared with the baseline in one group. Thus, the improvement in microalbuminuria might be due to the class effect of febuxostat rather than the absolute effect of serum urate levels, as also suggested by the findings of the study on benziodarone [39]. Microalbuminuria thus showed an improvement with febuxostat in patients with gout. A statistically significant change was seen only after 6 months of treatment with febuxostat, whereas more rapid improvement at 3 months, as expected, was seen with concomitant use of ARB. However, it is not clear whether ULT is indicated for patients with conditions like cardiovascular disease with microalbuminuria but without CKD or gout.

## 5. Conclusion

Urate-lowering therapy reduces the urinary microalbumin levels in the patients with gout.

## 6. Limitations

The major limitation of this study was the study being conducted in a single center. Likewise, other urate-lowering agents were not used and compared with febuxostat.

## Figures and Tables

**Table 1:** Baseline characteristics of the subjects (*n* = 114).

	ARB group (*n* = 35)	Non-ARB group (*n* = 79)	*p* value
Age (years) mean ± SD	51.4 ± 12.4	50.9 ± 12.8	0.847
Male/female (*n*)	33/2	72/7	
BMI (kg/m^2^) mean ± SD	25.6 ± 5.8	27.5 ± 4.9	0.082
Serum urate (mg/dL) mean ± SD	6.9 ± 2.4	7.5 ± 1.9	0.183
SBP (mmHg) mean ± SD	135.5 ± 23.6	126.4 ± 18.2	0.324
DBP (mmHg) mean ± SD	89.1 ± 14.2	86.5 ± 9.4	0.035
DM (%)	9.1	15.2	
Thyroid disease (%)	12.1	3.8	
Hyperlipidemia (*n*)	54.5	59.5	
MAU (mg/L) mean ± SD	127.9 ± 111.6	69.6 ± 14.6	0.056
CRP (mg/L) median	5.0	8.0	0.696
Waist circumference (cm)	98.0 ± 8.5	99.4 ± 9.7	0.674
TG (mg/dL) mean ± SD	286.6 ± 278.8	197.9 ± 122.1	0.011
HDL (mg/dL) mean ± SD	42.7 ± 10.0	46.3 ± 12.6	0.140
RBS (mg/dL) mean ± SD	116.6 ± 44.8	113.3 ± 42.7	0.747
Alcohol consumption (*n*)	8	22	
Smoking (*n*)	4	6	

BMI: body mass index; SBP: systolic blood pressure; DBP: diastolic blood pressure; ARB: angiotensin receptor blocker; MAU: urinary microalbumin; RBS: random blood sugar; CRP: C-reactive protein; LDL: low-density lipoprotein; HDL: high-density lipoprotein; TCH: total cholesterol; TG: triglyceride.

**Table 2:** Change in MAU and SUA after 3 and 6 months of treatment in ARB group (*n* = 35).

	Baseline	3 months	*p* value^∗^	6 months	*p* value^∗^
Urinary microalbumin (MAU)	127.9 ± 111.6	72.0 ± 109.3	0.02	44.9 ± 61.6	0.07
Serum uric acid (SUA)	6.9 ± 2.4	5.8 ± 2.43	0.038	5.3 ± 2.2	0.348

^∗^Paired *t*-test.

**Table 3:** Change in MAU and SUA after 3 and 6 months of treatment in non-ARB group (*n* = 79).

	Baseline	3 months	*p* value^∗^	6 months	*p* value^∗^
Urinary microalbumin (MAU)	69.6 ± 14.6	42.2 ± 54.4	0.14	15.7 ± 4.9	0.04
Serum uric acid (SUA)	7.5 ± 1.9	6.2 ± 2.2	0.082	5.7 ± 2.5	0.70

^∗^Paired *t*-test.

## Data Availability

The data used to support the findings of this study are available from the corresponding author upon request.
